# Reemergence of Yellow Fever in Brazil: The Role of Distinct Landscape Fragmentation Thresholds

**DOI:** 10.1155/2021/8230789

**Published:** 2021-07-23

**Authors:** Roberto C. Ilacqua, Antônio R. Medeiros-Sousa, Daniel G. Ramos, Marcos T. Obara, Walter Ceretti-Junior, Luis F. Mucci, Mauro T. Marrelli, Gabriel Z. Laporta

**Affiliations:** ^1^Setor de Pós-graduação, Pesquisa e Inovação, Centro Universitário FMABC, Fundação ABC, Santo André, SP, Brazil; ^2^Departamento de Epidemiologia, Faculdade de Saúde Pública da Universidade de São Paulo (FSP-USP), Cerqueira César, SP, Brazil; ^3^Coordenação Geral de Vigilância de Arboviroses, Ministério da Saúde (MS), Brasília, DF, Brazil; ^4^Faculdade de Ceilândia, Universidade de Brasília (UNB), Brasília, DF, Brazil; ^5^Superintendência de Controle de Endemias (SUCEN), Secretaria de Estado da Saúde, Taubaté, SP, Brazil

## Abstract

Yellow Fever Virus (YFV) reemergence in Brazil was followed by human suffering and the loss of biodiversity of neotropical simians on the Atlantic coast. The underlying mechanisms were investigated with special focus on distinct landscape fragmentation thresholds in the affected municipalities. An ecological study in epidemiology is employed to assess the statistical relationship between events of YFV and forest fragmentation in municipal landscapes. Negative binomial regression model showed that highly fragmented forest cover was associated with an 85% increase of events of YFV in humans and simians (*RR* = 1.85, CI 95% = 1.24–2.75, *p*=0.003) adjusted by vaccine coverage, population size, and municipality area. Intermediate levels of forest cover combined with higher levels of forest edge densities contribute to the YFV dispersion and the exponential growth of YF cases. Strategies for forest conservation are necessary for the control and prevention of YF and other zoonotic diseases that can spillover from the fragmented forest remains to populated cities of the Brazilian Atlantic coast.

## 1. Introduction

Reemergence of Yellow Fever Virus (YFV) has been reported in the extra-Amazonian region of Brazil since the 2000s [1–4]. However, the ongoing YFV reemergence which started in 2014 has been resulting in widespread virus dissemination and an extended transmission period [5–7]. The transmission zone has expanded from the endemic hub of the disease in the Amazon to the Brazilian Atlantic coast where the virus had not been recorded for more than 60 years [8, 9]. The expansion of the transmission zone has been driving the increase of YFV vaccine coverage to nonendemic territories [4]. Thousands of cases and deaths are occurring, causing impacts on public health and on the biodiversity of neotropical primates [10]. The most affected nonhuman primate species are New World Monkeys of the genera *Callithrix* (marmosets) and *Alouatta* (howler monkeys) [10–12]. Higher male frequency (80%), average age of 50 years, and residence in rural areas are the main characteristic of human cases [13]. In 2020, 881 suspected human cases occurred in southern Brazilian states, from which 18 nonvaccinated men between 18 and 59 years old have been confirmed as YF cases [14]. The dispersion of YFV in the landscape of cities on the Atlantic coast follow ecological corridors through the fragmented forest remains [15]. Forest fragments in the urbanized settings constitute structural landscape pathways for the circulation of nonhuman primates (howler monkeys and marmosets) and sylvatic mosquitoes with some level of synanthropic behavior (*Haemagogus leucocelaenus* as primary vector and *Aedes serratus*, *Psorophora ferox*, and *Aedes scapularis* as auxiliary vectors) [16–19], thus enabling YFV to reach out nonendemic territories [20].

YFV dispersion to nonendemic territories lacking vaccination or coverage can help in the increase of disease incidence [4]. In 2014–2019 a total of 4,217 nonhuman primate deaths and 852 human deaths from 2,839 human cases were confirmed [4, 7, 10, 14]. Urban transmission of YFV by *Aedes aegypti* was mostly feared in metropolitan areas. However, urban YF transmission during the YFV reemergence in Brazil has not been confirmed by the Brazilian Ministry of Health [4, 10]. The spatiotemporal overlap between epizootics in nonhuman primates and human cases, the demographic profile of human cases, and the absence of evidence on the participation of *Ae*. *aegypti* showed that YFV transmission is more likely to occur on the forest edges [4, 10].

Challenges for YF prevention and control include the understanding of the stability of endemic transmission in the Amazon region and mechanisms of dispersion to the nonendemic area (extra-Amazonian foci). Although land use land cover approaches are often applied to studies of zoonotic diseases in Atlantic Forest [21, 22], they are rarely applied to understand the mechanisms underlying the reemergence of YFV in Brazil (but see [23, 24]). In the present work, we tested the effect of landscape fragmentation thresholds on this reemergence. The goal here was to assess the relationship between distribution of YF cases and the associated local forest fragmentation.

## 2. Materials and Methods

### 2.1. Study Area and Design

**Figure 1:**
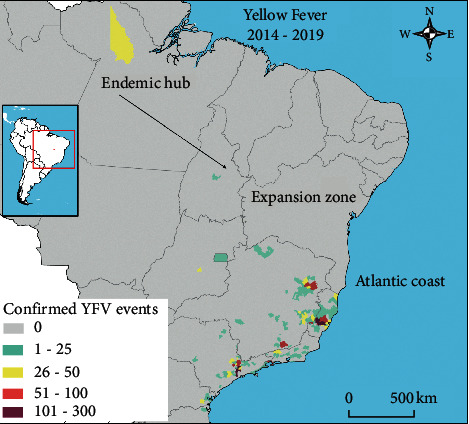
Distribution of the number of laboratory confirmed YFV in humans and nonhuman primates, Brazilian municipalities, 2014–2019.

This is an ecological study in which the population aggregate is the municipality reporting YF in Brazil, 2014–2019. YF events in humans and nonhuman primates were obtained from the General Coordination of Arbovirus Surveillance of the Ministry of Health, via the Law of Access to Information, protocol no. 25820004039202025. The eligibility criteria were (1) laboratory confirmation of events in humans and animals; and (2) selection of municipalities with both events occurring at the reemergence period (2014–2019). All the eligible municipalities and their associated number of events are shown (Figure 1).

The sample size of this study consisted of the total number of eligible municipalities, which resulted in *N* = 151 (Figure 1). The response variable (*y*) was the reemergence of YFV—the sum of confirmed YFV events in humans and animals in all these municipalities. The explanatory variables (*X*_n_) were ecological and environmental determinants of the reemergence of YFV—the proportion of remaining forest cover (%) and the density of forest edge (m/ha) in the municipality [15, 16, 20]. Using the front view of the municipality landscape, these determinants were estimated using remote sensing images and a supervised classification approach previously published by our group [25].

### 2.2. Estimation of Forest Cover and Forest Edge Density

Landsat-8 satellite OLI sensor images referring to the 151 selected municipalities were obtained between January 2018 to December 2019. The basic scenes representing 170 × 180 km of the Earth were downloaded from the *Glovis* portal supported by the US Geological Survey [26]. From basic scenes, the satellite bands 2-blue, 3-green, 4-red, 5-near infrared, and 6, 7-short wave infrared were used. These bands were digitally processed in QGIS software v. 3.4 *Madeira*.

The first digital processing was the correction of atmospheric interference in these bands with the reflectance algorithm of SCP plugin v. 7.7.1 [27]. The administrative area of the municipality [28] was clipped from within each scene after the reflectance stage. Bands 4-3-2 (natural color), 5-4-3 (infrared), and 6-5-4 (false color) were stacked to obtain composite images. These composite images were used for the supervised classification of municipality landscape.

Supervised classification of municipality landscape was employed to generate municipal land use land cover using the same approach previously published by Ilacqua et al. [25]. Three classes of land use land cover were estimated: (1) Preserved Forest (green)—native remnants of preserved forest; (2) Exposed Soil (yellow)—set of urban and rural features; (3) Ground Waters (blue)—surface waters.

The quantification of the area and the edge (perimeter) of the class Preserved Forest was carried out with algorithms in the software *Fragstats* v. 4.2, as follows:

(1) $$\begin{array}{c} P_{\text{forest}=\left(\sum^{}\text{forest area}\left(m^{2}\right)/\text{municipality area}\left(m^{2}\right)\right)\;100}, \end{array}$$

where the percentage of forest cover (*P*_forest_ = 0–100%) is the sum of all forest areas divided by the total area of the municipality, multiplied by 100 to convert into percentual.

(2) $$\begin{array}{c} ED_{\text{forest}=\left(\sum^{}\text{edges of forest}\left(m\right)/\text{municipality area}\left(m^{2}\right)\right)10.000}, \end{array}$$

where the edge density of forest (meters per hectare) is the sum of the length of all forest edges divided by the total area of the municipality, multiplied by 10,000 to convert to hectare.

### 2.3. Data Analysis

Descriptive statistics of the number of confirmed YFV events was done per age and proportion of male gender in human cases and proportion of nonhuman primate species (marmosets and howler monkeys) in epizootics. These variables were categorized according to the categories of greatest risk of exposure [11, 13], as follows:

1. Age, 60–30 years (exposure risk) and >60 years or <30 years (baseline)
2. Male proportion, 85–55% (exposure risk) and >85% or <55% (baseline)
3. Proportion of marmosets and howler monkeys, 0–20% (baseline), 21–60% (exposure risk 1), and 61–100% (exposure risk 2)

The relationship between forest edge density and forest cover was estimated using a second-order linear model:

(3) $$\begin{array}{c} \text{edge density}=\text{cover}+{\text{cover}}^{2}, \end{array}$$

where the forest edge density is a function of forest cover.

Distinct landscape fragmentation thresholds [29–31] were based on equation (3): (1) baseline = 0, <30% or >70% of forest cover and <80 (m/ha) of forest edge density; (2) partially fragmented forest (=1), 30–70% forest cover and <80 (m/ha) of forest edge density; and (3) highly fragmented forest (=2), 30–70% forest cover and ≥80 (m/ha) of forest edge density.

The relative risk (*RR*) of YFV reemergence in function of landscape fragmentation categories was estimated using a negative binomial regression model, as follows:

(4) $$\begin{array}{c} \ln\left(\mu_{i}\right)=\;\beta_{0}+\beta_{1}\text{Frag}1_{i}+\beta_{2}\text{Frag}2_{i}+\beta_{3}{\text{Vaccine}}_{i}+\beta_{4}{\text{Population}}_{i}+\beta_{5}{\text{Area}}_{i}, \end{array}$$

where Frag1 = partially fragmented forest, Frag2 = highly fragmented forest, Vaccine = vaccination coverage, Population = municipality population, and Area = municipality area.

Vaccine, Population, and Area were used to adjust the *RR* of YFV reemergence in function of forest fragmentation. The invasion of YFV to territories with no vaccination coverage was decisive for the exponential growth of new cases. Considering the territorial vaccine coverage prior to the reemergence of YFV, Vaccine was classified into exposure category (no vaccination coverage or recommendation) and baseline (vaccination coverage) [4]. The larger the population or the area of the municipality, the greater the number of cases. Population was obtained from the 2018 municipal population projection and was classified into large (exposed) if >100,000 people or as small/medium-sized (nonexposed) if ≤100,000 people [28]. Area [28] was classified into exposed if >50,000 m^2^ or nonexposed if ≤50,000 m^2^.

The adjusted-*RR* was calculated as the exponential *β* value estimated by the negative binomial regression equation. We tested the following null hypothesis (*H*_0_: *RR* = 1) with its alternative (*H*_a_: *RR* ≠ 1) considering 0.05 (type-I error) as the level of significance (*α*) and (1 − *α*) % as the confidence interval. *RR* > 1 meant a reciprocal association between YFV reemergence and forest fragmentation, while *RR* < 1 meant that this relationship was not reciprocal. Finally, if *RR* = 1 it was assumed null effect.

## 3. Results

The total number of human and nonhuman primate cases in the selected municipalities that had both events was 3,541. The average number of events per municipality (*N* = 151) was 24 (±36) (range, 2–268). This variable (number of events) did not adhere to the Gaussian distribution and presented overdispersion in relation to the Poisson distribution (see Table S1 in the Supplementary Material for comprehensive dataset visualization).

The average number of YF events was 2.8 times higher when human cases had male proportion between 55 and 85%. It was 1.7 times higher when human cases had average age between 30 and 60 years. It was 1.5 and 1.8 times higher when nonhuman primate cases had proportion of marmosets and howler monkeys of 61–100% and 21–60%, respectively.

**Figure 2:**
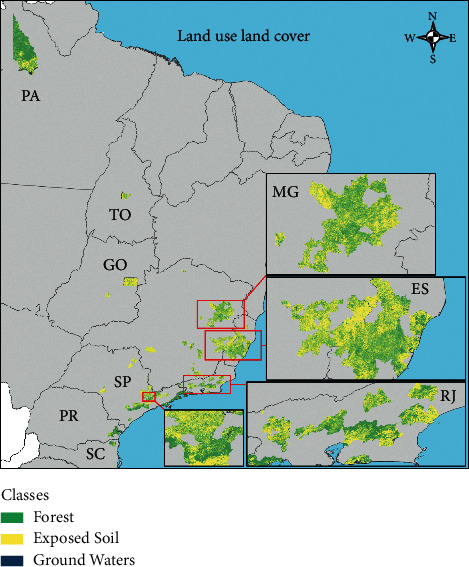
Forest cover and configuration in municipal landscape of the municipalities reporting YFV in humans and animals during reemergence.

The average forest cover (%) among these municipalities was 44% (±18%, min-max = 7–95%) and the average forest edge density (m/ha) was 73 (±21, min-max = 21–116) (Figure 2).

**Figure 3:**
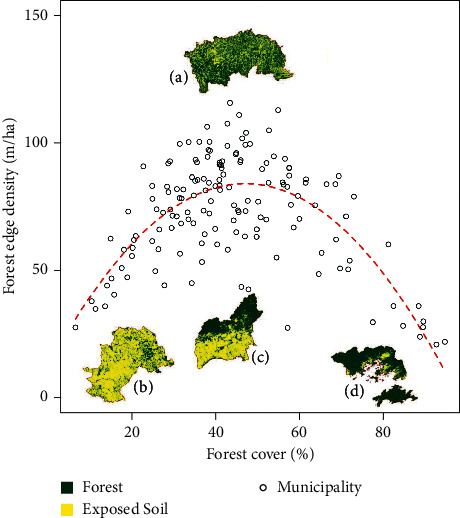
Density of forest edge due to forest cover. The red dotted line represents the fit curve of the linear model. (a) Domingos Martins-ES, an example of a municipality with maximum fragmentation and forest cover 70–30%. (b) Campinas-SP, an example of a municipality with low fragmentation and forest cover <30%. (c) Guarulhos-SP, an example of a municipality with low fragmentation and forest coverage 70–30%. (d) Angra dos Reis-RJ, example of a municipality with low fragmentation and forest cover >70%.

The linear model equation between forest edge density (*y*) and forest cover (*x*) was *y* = −0.03*x*^2^ + 3.1*x* + 12.3, with an adjusted *R*^2^ of 50% (*F*_2,148_ = 78, *p* < 0.001). According to the equation, forest fragmentation in the landscape was greater in municipalities with intermediate forest cover (30–70%), while forest fragmentation was lower in deforested (<30% forest cover) and preserved (>70% forest cover) landscapes (Figure 3).

Highly fragmented municipalities (Figure 3(a)) have an 85% higher risk of YF disease occurrence, compared to the category with the lowest exposure (Figures 3(b) and 3(d)). There was no difference for partially fragmented municipalities (Figure 3(c)). The relative risk values were adjusted by vaccination coverage, population, and municipality area (Table 1).

## 4. Discussion

The relationship between forests and diseases is complex [30]. On one hand, forests are home to many organisms and microorganisms that can cause zoonotic diseases in humans. On the other hand, forests also provide an important ecosystem service, known as zoonotic disease regulation [32]. If forests are preserved and a large part of their diversity is maintained, they have a low chance of transmitting zoonoses to humans or causing outbreaks, even if they harbor a high diversity of pathogens [33]. Zoonotic diseases are favored when humans break the rules of coexistence in equilibrium with the natural world [34]. Among the main human actions that lead to an increase in the incidence of zoonoses, deforestation and fragmentation of natural environments can be mentioned [22]. Deforestation carried out in the broad and unrestricted way in tropical forests is far from being just a moral problem―it is about to become a public health problem [35]. Studies have already shown that deforestation can lead to increased transmission of malaria [36], hantavirus [22], visceral leishmaniasis [37], and Chagas disease [38], to name just a few known examples. In the present study, we obtained evidence that accumulated deforestation between 30 and 70% and the fragmentation of the remaining forest equal to or above 80 m/ha, on the municipality scale, are associated with the occurrence of YF in Brazil, 2014–2019.

Upon reaching the Atlantic Forest in 2015 [39], YFV found a perfect scenario for its expansion, because the biome currently contains only 28% of its original cover in a highly fragmented state [40]. This virus was restricted to the Amazon biome for several years–the natural habitat, where the YF incidence is low due to high vaccination coverage. However, it has appeared in other regions of the country since 2008, causing outbreaks in humans and decimating entire populations of nonhuman primates [41, 42]. In these highly fragmented landscapes of the Atlantic Forest, there is low diversity of vertebrate species—a direct consequence of deforestation, forest fragmentation, and hunting [43]. When few species are left in the ecosystem, those becoming extremely abundant have the potential for amplifying novel pathogen lineages.

Among abundant species in fragmented landscapes are howler monkeys [44], hosts that can amplify lineages of YFV [11, 12] and are frequently bitten by mosquito vector species in the forest [45, 46]. Viral amplification in this transmission expansion scenario has been responsible for the occurrence of several human deaths and the local extinction of nonhuman primates [5, 6, 39]. These outcomes in the fragments of the Atlantic Forest confirm that the relationship between forests and diseases can be challenging for human persistence. We will play “Russian roulette” if we continue to deforest and put pressure on wild cycles in this way [47]. Every year the number of cases of zoonoses grows in Brazil [48], and soon we will be able to produce pandemics like that of SARS-CoV-2 as a result of our actions.

There is a theoretical study in the Atlantic Forest that shows the amount of forest cover remnant sufficient for providing zoonotic disease regulation service. This amount, called the critical threshold, would be around 30% of forest cover [43]. Below this value, the loss of connectivity between the remaining fragments would be severely damaged; that is, the landscape would be formed by innumerable portions of forest very isolated from each other. As a result, the animals and plants that inhabit these landscapes would be more prone to extinction and there would be a disproportionate loss of species. The species that end up managing to survive in such degraded landscapes are precisely those considered hosts and vectors of diseases, which, without competition and predators, end up increasing in abundance and becoming dominant. This theoretical model was tested in rural environments dominated by species of small mammalian reservoirs of hantavirus [21, 22]. Landscapes with forest cover values below 30% had less species diversity and greater reservoir abundance [22]. However in the present study we found that values above 30% of forest cover (up to 70%) and high fragmentation (Figure 3(a)) are associated with the risk of YFV reemergence in the Atlantic Forest. The conservation of preserved municipalities (Figure 3(d)) and the restoration of native forest in degraded municipalities (Figure 3(b)) into municipalities with low fragmentation (Figure 3(c)) are recommendations of this study to maintain the service of regulation of YFV and increase biodiversity in the Atlantic Forest. The maintenance of the virus in the Amazon region with endemic occurrence, that is, stable low incidence transmission, is favored by disease dilution effect mechanism [32] due to high forest cover (Figure 2, Pará state), in conjunction with high vaccination coverage.

Brazil has environmental legislation, the Forest Code, which protects about 50% of the native vegetation present in the country [49]. The law obliges landowners to keep part of their farms covered with native vegetation (the so-called legal reserves) to conserve biodiversity and the provision of ecosystem services, including the regulation of zoonotic diseases [50]. However, most of these owners still need to restore forest areas to meet the requirements that the legislation applies [51]. To prevent the emergence of new outbreaks, the maintenance of the Forest Code and the restoration of all vegetation that is in deficit by the law are essential. A recent study showed, for example, that if the Atlantic Forest was restored until the requirements required by the law were met—which represents a restoration of almost 6 million hectares of forest—the abundance of hantavirus reservoirs would decrease by up to 90%, benefiting about 2.8 million people in the region [22]. In addition to respecting the Forest Code, it is essential to reduce deforestation. In the present study, it was evidenced that, in addition to the 30% forest cover [43], the forest fragmentation must be kept low (<80 m/ha) in the municipality to allow the regulation of YFV. Thus, when deforesting and fragmenting an environment, the ideal would be to do so to respect these limits, managing landscapes so that they have a low risk of transmission and can still be used by humans. Conservation units also play a key role in stopping the transmission of zoonoses. Thus, public policies aimed at complying with current environmental legislation, respect for existing conservation units, and the forest restoration required by the Forest Code would be sufficient to prevent a new pandemic.

## 5. Conclusions

The role of distinct landscape fragmentation thresholds on the reemergence of YFV in Brazil was tested. The stability of endemic foci in the Amazon region is based on forested preserved municipal landscapes in which transmission cycles occur periodically and in equilibrium. Intermediate forest cover and a high level of forest edges (forest fragmentation) are mechanisms of YFV dispersion and exponential growth of cases in the municipal landscape of the Atlantic coast. Strategies for forest conservation are necessary for the control and prevention of YF and other zoonotic diseases that can spillover from the fragmented forest remains to populated cities of the Brazilian Atlantic coast.

## Figures and Tables

**Table 1:** Association between YF occurrence and forest fragmentation in negative binomial regression.

*X*_n_ (independent variables)	Adjusted relative risk	Confidence interval 95%	*p*
High forest fragmentation	1.85	1.24–2.75	0.003^1^
Partial forest fragmentation	0.81	0.54–1.23	0.23
No vaccination or coverage	1.74	1.25–2.42	0.001^1^
Larger municipal population (>100,000 ppl)	1.37	0.93–2.01	0.11
Larger municipal area (>50,000 m^2^)	1.61	1.17–2.21	0.004^1^

^1^Statistically significant variables at the confidence level of 0.05.

## Data Availability

The data used are given as supplementary material.

## Supplementary Materials

Supplementary Materials Table S1: the dataset used for analysis.
