# Supplementary material for: Reemergence of Yellow Fever in Brazil: The Role of Distinct Landscape Fragmentation Thresholds
Source: J Environ Public Health. 2021 Jul 23;2021:8230789. doi: 10.1155/2021/8230789 (PMC8325590; doi:10.1155/2021/8230789)
Supplement: Supplementary Materials — Table S1: the dataset used for analysis. [file 8230789.f1.docx]

# Journal of Environmental and Public Health

**Re-emergence of yellow fever in Brazil: the role of distinct landscape fragmentation thresholds**

Roberto C. Ilacqua^1^, Antônio R. Medeiros-Sousa^2^, Daniel G. Ramos^3^, Marcos T. Obara^4^, Walter Ceretti-Junior^2^, Luis F. Mucci^5^, Mauro T. Marrelli^2^, Gabriel Z. Laporta^1^

^1^Setor de Pós-graduação, Pesquisa e Inovação, Centro Universitário da Faculdade de Medicina do ABC (FMABC), Fundação ABC, Santo André, SP, Brazil

^2^Departamento de Epidemiologia, Faculdade de Saúde Pública da Universidade de São Paulo (FSP-USP), São Paulo, SP, Brazil

^3^Coordenação Geral de Vigilância de Arboviroses, Ministério da Saúde (MS), Brasília, DF, Brazil

^4^Faculdade de Ceilândia, Universidade de Brasília (UNB), Brasília, DF, Brazil

^5^Superintendência de Controle de Endemias (SUCEN), Secretaria de Estado da Saúde, Taubaté, SP, Brazil

Correspondence should be addressed to Gabriel Z. Laporta; gabriel.laporta@fmabc.br

Table S1 – Dataset utilized for data analysis (*N*=151 municipalities).

| **state** | **municipality** | **cases** | **area** | **pop** | **forest** | **Edforest** | **Pmasc** | **age** | **Pmonkey** | **vaccine** |
| --- | --- | --- | --- | --- | --- | --- | --- | --- | --- | --- |
| TO | Palmas | 15 | 222249 | 291855 | 31.4243 | 56.5208 | 40 | 29 | 100 | 0 |
| SP | Águas da Prata | 5 | 14296.05 | 8137 | 44.2771 | 69.5458 | 100 | 35.36 | 0 | 0 |
| SP | Atibaia | 51 | 47844.63 | 141398 | 40.9314 | 83.3153 | 76.47 | 44.6 | 92.68 | 1 |
| SP | Batatais | 2 | 85037.4 | 62024 | 14.8496 | 62.3944 | 100 | 39 | 100 | 0 |
| SP | Bragança Paulista | 74 | 51248.52 | 166753 | 30.6996 | 81.9044 | 100 | 65 | 71.23 | 1 |
| SP | Caieiras | 16 | 9763.65 | 100129 | 69.7175 | 50.7013 | 100 | 49.5 | 14.29 | 1 |
| SP | Campinas | 46 | 79478.46 | 1194094 | 17.5297 | 50.8957 | 100 | 64 | 88.89 | 1 |
| SP | Caraguatatuba | 8 | 48657.42 | 119625 | 77.524 | 29.6002 | 100 | 26 | 100 | 1 |
| SP | Cotia | 10 | 32406.48 | 244694 | 58.5788 | 66.8061 | 88.89 | 39.11 | 100 | 1 |
| SP | Embu-Guaçu | 10 | 25560.37 | 68856 | 56.2105 | 83.6972 | 100 | 49.44 | 100 | 1 |
| SP | Franco da Rocha | 8 | 13277.79 | 152433 | 47.6964 | 77.238 | 100 | 38 | 100 | 1 |
| SP | Guarulhos | 28 | 31859.46 | 1365899 | 47.8186 | 42.4583 | 88.89 | 43.93 | 100 | 1 |
| SP | Ibiúna | 43 | 105852.8 | 78262 | 68.7528 | 62.0336 | 92.86 | 50.21 | 100 | 1 |
| SP | Igaratá | 4 | 29273.4 | 9483 | 49.7605 | 63.0818 | 100 | 57.33 | 100 | 1 |
| SP | Itatiba | 20 | 322227.2 | 11909 | 29.6962 | 71.3993 | 100 | 72 | 66.67 | 1 |
| SP | Jarinu | 31 | 20753.46 | 29456 | 40.9109 | 92.2617 | 90 | 45.95 | 90.48 | 1 |
| SP | Joanópolis | 2 | 37412.37 | 13098 | 54.4866 | 55.0262 | 100 | 51 | 0 | 1 |
| SP | Jundiaí | 85 | 43123.59 | 41481 | 52.0379 | 69.9258 | 100 | 55 | 86.9 | 1 |
| SP | Mairiporã | 268 | 32065.02 | 98374 | 69.4197 | 86.9798 | 79.17 | 43.61 | 70 | 1 |
| SP | Monte Alegre do Sul | 39 | 11028.78 | 7964 | 40.8766 | 85.5027 | 83.33 | 48.83 | 42.42 | 1 |
| SP | Nazaré Paulista | 6 | 32609.34 | 18346 | 59.9974 | 70.1403 | 100 | 57 | 0 | 1 |
| SP | Pariquera-Açu | 9 | 35996.22 | 19570 | 57.06 | 82.797 | 85.71 | 42.14 | 100 | 1 |
| SP | Piedade | 8 | 74738.34 | 55149 | 50.1217 | 76.8935 | 83.33 | 39.74 | 100 | 1 |
| SP | Ribeirão Preto | 2 | 65173.86 | 694534 | 6.4978 | 27.6226 | 100 | 53 | 0 | 0 |
| SP | Salto de Pirapora | 2 | 28132.02 | 44972 | 38.05 | 94.5087 | 100 | 12 | 100 | 1 |
| SP | Santa Cruz do Rio Pardo | 5 | 111468.2 | 47395 | 15.7044 | 40.3724 | 100 | 17 | 25 | 0 |
| SP | São José dos Campos | 11 | 109867.7 | 713943 | 36.7019 | 53.2928 | 77.78 | 34.67 | 100 | 1 |
| SP | São Lourenço da Serra | 5 | 18647.64 | 15667 | 81.2139 | 60.0816 | 100 | 19 | 100 | 1 |
| SP | São Miguel Arcanjo | 6 | 93171.6 | 32859 | 44.9303 | 64.8874 | 100 | 40 | 100 | 0 |
| SP | São Paulo | 200 | 152095.8 | 12176866 | 34.266 | 44.9602 | 69.7 | 43.04 | 95.81 | 1 |
| SP | São Sebastião | 8 | 39929.21 | 87596 | 89.5526 | 27.7177 | 100 | 27.33 | 100 | 1 |
| SP | Serra Negra | 2 | 20372.76 | 29001 | 34.3213 | 86.5332 | 100 | 58 | 100 | 1 |
| SP | Socorro | 4 | 44892.18 | 40648 | 18.0857 | 58.1306 | 0 | 53 | 0 | 1 |
| SP | Tapiraí | 5 | 75564.17 | 7850 | 92.666 | 20.8326 | 0 | 50 | 100 | 1 |
| SP | Taubaté | 5 | 62388.52 | 311854 | 25.5921 | 49.6364 | 100 | 40 | 100 | 1 |
| SP | Tuiuti | 26 | 12670.02 | 6808 | 19.9726 | 55.6574 | 100 | 56 | 48 | 1 |
| SP | Ubatuba | 13 | 72475.65 | 89747 | 88.7718 | 23.9555 | 90.91 | 50.73 | 100 | 1 |
| SP | Valinhos | 9 | 14860.08 | 127123 | 28.3723 | 82.869 | 100 | 32.71 | 50 | 1 |
| SC | Joinville | 2 | 112637.2 | 583144 | 71.5814 | 50.3482 | 100 | 36 | 0 | 1 |
| RJ | Angra dos Reis | 80 | 82372.59 | 200407 | 84.7493 | 28.2349 | 68.19 | 39.88 | 100 | 1 |
| RJ | Barra do Piraí | 12 | 57865.86 | 99969 | 34.609 | 68.4637 | 72.72 | 41.45 | 100 | 1 |
| RJ | Cachoeiras de Macacu | 5 | 95434.2 | 58560 | 65.3069 | 56.8667 | 66.67 | 61 | 100 | 1 |
| RJ | Duas Barras | 14 | 37544.04 | 11454 | 45.355 | 73.4705 | 84.61 | 44.08 | 100 | 1 |
| RJ | Engenheiro Paulo de Frontin | 7 | 13289.04 | 13929 | 71.1234 | 71.1812 | 100 | 49.83 | 100 | 1 |
| RJ | Guapimirim | 3 | 36083.97 | 59613 | 44.386 | 75.5646 | 100 | 25 | 50 | 1 |
| RJ | Itatiaia | 6 | 24495.21 | 31537 | 64.6091 | 48.525 | 50 | 38.25 | 50 | 1 |
| RJ | Macaé | 6 | 121868.2 | 251631 | 37.3403 | 64.1301 | 60 | 50.4 | 100 | 1 |
| RJ | Mangaratiba | 4 | 35614.98 | 43689 | 82.4445 | 35.9604 | 100 | 11.5 | 100 | 1 |
| RJ | Maricá | 14 | 36275.22 | 157789 | 39.8697 | 66.2154 | 33.33 | 40 | 27.27 | 1 |
| RJ | Miguel Pereira | 7 | 28913.76 | 25493 | 41.018 | 81.6016 | 100 | 42.8 | 100 | 1 |
| RJ | Paraty | 12 | 92486.97 | 42630 | 88.4934 | 35.9454 | 60 | 50.8 | 100 | 1 |
| RJ | Petrópolis | 5 | 79588.35 | 305687 | 63.7316 | 75.4912 | 100 | 55.75 | 100 | 1 |
| RJ | Piraí | 4 | 50514.03 | 28999 | 45.4942 | 72.7723 | 66.67 | 56.67 | 100 | 1 |
| RJ | Santa Maria Madalena | 2 | 81595.08 | 10417 | 42.861 | 58.0895 | 100 | 42 | 0 | 1 |
| RJ | Silva Jardim | 6 | 93963.06 | 21773 | 46.1564 | 43.436 | 100 | 46.6 | 0 | 1 |
| RJ | Sumidouro | 14 | 39577.05 | 15577 | 44.7898 | 95.5107 | 76.92 | 54.62 | 100 | 1 |
| RJ | Valença | 35 | 130424.1 | 76163 | 26.5421 | 66.0025 | 81.82 | 44.48 | 100 | 1 |
| RJ | Volta Redonda | 8 | 18236.88 | 271998 | 29.4786 | 66.6101 | 100 | 41 | 100 | 1 |
| PR | Antonina | 5 | 88277.49 | 21933 | 89.6014 | 29.8619 | 100 | 27 | 100 | 1 |
| PR | Morretes | 3 | 68481 | 16366 | 94.7131 | 21.914 | 100 | 64.5 | 100 | 1 |
| PR | Paranaguá | 2 | 82730.61 | 153666 | 57.153 | 27.4426 | 0 | 10 | 100 | 1 |
| PR | São José dos Pinhais | 27 | 94655.43 | 317476 | 57.9308 | 75.6914 | 0 | 62 | 100 | 1 |
| PA | Alenquer | 49 | 2362774 | 56480 | 72.9638 | 78.949 | 100 | 14.5 | 0 | 0 |
| PA | Monte Alegre | 30 | 1817603 | 57900 | 71.939 | 53.835 | 100 | 22.67 | 3.7 | 0 |
| MG | Água Boa | 2 | 132193.1 | 13600 | 48.199 | 99.6542 | 100 | 33 | 100 | 0 |
| MG | Aimorés | 14 | 134946.4 | 25193 | 11.2301 | 34.7814 | 60 | 45.4 | 0 | 0 |
| MG | Alvarenga | 9 | 27842.94 | 3973 | 31.3968 | 99.608 | 100 | 39.5 | 0 | 0 |
| MG | Antônio Carlos | 5 | 52970.31 | 11432 | 37.0733 | 73.3832 | 100 | 52.4 | 66.67 | 0 |
| MG | Belo Horizonte | 18 | 33122.25 | 2501576 | 14.0037 | 43.9825 | 100 | 79 | 70.59 | 0 |
| MG | Bom Jesus do Galho | 17 | 59293.53 | 15010 | 35.1523 | 93.3871 | 66.67 | 56.48 | 36.36 | 0 |
| MG | Caputira | 3 | 18794.88 | 9287 | 35.5056 | 92.3805 | 100 | 52 | 0 | 0 |
| MG | Caraí | 4 | 124529 | 23586 | 57.5405 | 90.2787 | 100 | 31 | 100 | 0 |
| MG | Caratinga | 44 | 126065.2 | 91503 | 19.0624 | 73.0584 | 100 | 33.5 | 97.62 | 0 |
| MG | Chalé | 5 | 21289.68 | 5709 | 29.2025 | 73.8189 | 100 | 45 | 0 | 0 |
| MG | Conceição de Ipanema | 8 | 25423.65 | 4570 | 37.539 | 81.4203 | 100 | 58 | 0 | 0 |
| MG | Durandé | 14 | 21776.31 | 7811 | 53.8595 | 94.7475 | 50 | 42.5 | 0 | 0 |
| MG | Entre Folhas | 8 | 8532.54 | 5362 | 28.7134 | 80.6876 | 100 | 49.83 | 75 | 0 |
| MG | Esmeraldas | 2 | 90885.51 | 702 | 47.2651 | 104.0258 | 0 | 53 | 100 | 0 |
| MG | Felício dos Santos | 7 | 35764.11 | 4804 | 33.2252 | 72.7098 | 100 | 44 | 0 | 0 |
| MG | Franciscópolis | 23 | 71833.32 | 5446 | 54.855 | 112.9047 | 0 | 79 | 100 | 0 |
| MG | Frei Gaspar | 22 | 62720.91 | 5891 | 41.4066 | 90.0666 | 92.31 | 39.97 | 100 | 0 |
| MG | Imbé de Minas | 32 | 19706.04 | 6865 | 43.4087 | 115.7676 | 90.91 | 41.75 | 66.67 | 0 |
| MG | Inhapim | 6 | 85949.91 | 24204 | 38.5124 | 100.4019 | 100 | 59.48 | 100 | 0 |
| MG | Ipatinga | 10 | 16499.61 | 261344 | 37.1294 | 85.9675 | 50 | 60.5 | 100 | 0 |
| MG | Itambacuri | 21 | 142197.3 | 23212 | 45.9504 | 93.2672 | 76.92 | 47.31 | 75 | 0 |
| MG | Itueta | 19 | 45288.72 | 6039 | 20.1289 | 58.8305 | 100 | 44 | 0 | 0 |
| MG | Januária | 18 | 665671.1 | 67628 | 13.5632 | 35.8456 | 100 | 52.5 | 100 | 0 |
| MG | José Raydan | 7 | 18097.29 | 4938 | 45.5767 | 111.0315 | 83.33 | 50.5 | 100 | 0 |
| MG | Juiz de Fora | 55 | 143539.7 | 564310 | 30.624 | 71.1727 | 68.29 | 53.69 | 92.86 | 0 |
| MG | Ladainha | 68 | 86802.12 | 18026 | 52.5976 | 84.365 | 79.25 | 40.97 | 80 | 0 |
| MG | Lajinha | 21 | 43234.65 | 19928 | 24.8326 | 83.1847 | 70.69 | 42.79 | 0 | 0 |
| MG | Lima Duarte | 8 | 84809.34 | 16671 | 39.8797 | 83.0594 | 85.71 | 52.14 | 100 | 0 |
| MG | Malacacheta | 7 | 72900.72 | 18700 | 42.7813 | 98.864 | 75 | 48 | 100 | 0 |
| MG | Manhuaçu | 49 | 62922.24 | 89256 | 29.0763 | 92.8208 | 90.91 | 51.52 | 63.16 | 0 |
| MG | Mar de Espanha | 2 | 37169.55 | 12725 | 33.4624 | 70.0251 | 100 | 41 | 100 | 0 |
| MG | Mariana | 3 | 119413 | 60142 | 47.13 | 73.2443 | 50 | 67.5 | 100 | 0 |
| MG | Matipó | 7 | 26728.2 | 18808 | 20.4509 | 61.8437 | 100 | 38 | 0 | 0 |
| MG | Minas Novas | 6 | 181425.2 | 31471 | 22.6828 | 90.7572 | 33.33 | 49 | 0 | 0 |
| MG | Mutum | 37 | 125182.6 | 26997 | 24.8052 | 61.4285 | 100 | 47.67 | 0 | 0 |
| MG | Nova Lima | 5 | 42881.76 | 93577 | 47.0088 | 63.2318 | 100 | 51.5 | 33.33 | 0 |
| MG | Novo Cruzeiro | 41 | 170602.5 | 31326 | 61.4636 | 84.4062 | 91.18 | 44.75 | 100 | 0 |
| MG | Peçanha | 3 | 99757.53 | 17545 | 43.2007 | 82.5345 | 100 | 31 | 100 | 0 |
| MG | Piranga | 12 | 65877.75 | 17618 | 41.2205 | 91.2199 | 90.91 | 51.64 | 100 | 0 |
| MG | Poté | 29 | 62640.72 | 16491 | 44.9005 | 96.0588 | 80 | 50.35 | 100 | 0 |
| MG | Ressaquinha | 3 | 18451.89 | 4798 | 31.7889 | 78.2554 | 100 | 60 | 50 | 0 |
| MG | Sabará | 3 | 30206.07 | 135421 | 51.0352 | 89.7058 | 100 | 74 | 0 | 0 |
| MG | Santa Maria do Suaçuí | 8 | 62481.15 | 14620 | 42.7603 | 107.5942 | 100 | 46.33 | 0 | 0 |
| MG | Santa Rita de Minas | 4 | 6824.43 | 7155 | 28.6784 | 91.9286 | 100 | 46 | 0 | 0 |
| MG | Santa Rita do Itueto | 9 | 48546.27 | 5522 | 41.8046 | 87.6135 | 100 | 51.13 | 0 | 0 |
| MG | Santana do Manhuaçu | 13 | 34799.13 | 8681 | 36.0886 | 100.387 | 33.33 | 43.33 | 0 | 0 |
| MG | São João Evangelista | 8 | 47849.22 | 15781 | 32.2192 | 81.7236 | 100 | 51 | 16.67 | 0 |
| MG | São José do Jacuri | 24 | 34537.14 | 6477 | 38.6446 | 96.9122 | 50 | 47 | 0 | 0 |
| MG | São Roque de Minas | 19 | 209853.4 | 7026 | 10.3492 | 37.8857 | 0 | 20 | 16.67 | 0 |
| MG | Setubinha | 22 | 53540.91 | 12134 | 52.68 | 104.9789 | 83.33 | 42.12 | 100 | 0 |
| MG | Simonésia | 29 | 48733.2 | 19528 | 33.5034 | 100.33 | 58.33 | 40.58 | 0 | 0 |
| MG | Teófilo Otoni | 91 | 324611.7 | 140235 | 35.6371 | 85.5427 | 94.44 | 53.57 | 91.78 | 0 |
| MG | Ubaporanga | 4 | 18933.66 | 12449 | 24.8724 | 78.0372 | 100 | 51 | 0 | 0 |
| GO | Goiânia | 28 | 73395.72 | 1495705 | 18.8883 | 47.2617 | 64.71 | 63 | 45.45 | 0 |
| ES | Afonso Cláudio | 112 | 95185.8 | 30720 | 41.5523 | 92.7611 | 60 | 38.08 | 3.92 | 1 |
| ES | Alto Rio Novo | 3 | 22767.93 | 7798 | 31.3479 | 78.3905 | 100 | 23 | 0 | 1 |
| ES | Aracruz | 3 | 142325.7 | 99305 | 40.3366 | 60.0891 | 100 | 40 | 100 | 1 |
| ES | Baixo Guandu | 22 | 91732.05 | 30862 | 20.9061 | 63.4476 | 80 | 40.13 | 0 | 1 |
| ES | Brejetuba | 16 | 34437.51 | 12381 | 56.5332 | 93.7089 | 100 | 44.5 | 0 | 1 |
| ES | Cachoeiro de Itapemirim | 2 | 87856.2 | 207324 | 38.8405 | 77.1876 | 100 | 38 | 100 | 1 |
| ES | Cariacica | 27 | 27975.51 | 378603 | 49.791 | 66.0889 | 100 | 39.4 | 45.45 | 1 |
| ES | Castelo | 14 | 66438.54 | 37317 | 45.8579 | 92.3947 | 83.33 | 47 | 0 | 1 |
| ES | Colatina | 25 | 141669.5 | 121580 | 34.728 | 79.3873 | 90 | 43 | 60 | 1 |
| ES | Conceição do Castelo | 31 | 36945.27 | 12638 | 59.6264 | 79.185 | 85.71 | 47.88 | 0 | 1 |
| ES | Domingos Martins | 214 | 122847.6 | 33711 | 57.5351 | 87.1358 | 75 | 43.5 | 31.05 | 1 |
| ES | Guarapari | 31 | 59439.51 | 122982 | 50.7093 | 70.7817 | 100 | 68 | 73.33 | 1 |
| ES | Ibatiba | 72 | 24076.35 | 25732 | 38.2693 | 97.2868 | 81.25 | 47.75 | 0 | 1 |
| ES | Irupi | 22 | 18476.91 | 13226 | 46.3631 | 101.819 | 100 | 37.25 | 0 | 1 |
| ES | Itaguaçu | 23 | 53156.16 | 14109 | 40.9088 | 91.4648 | 75 | 48.75 | 10.53 | 1 |
| ES | Itarana | 52 | 29880.72 | 10619 | 27.4337 | 72.6217 | 80 | 51.51 | 8.51 | 1 |
| ES | Laranja da Terra | 13 | 45853.47 | 10961 | 47.0661 | 99.2758 | 75 | 50 | 0 | 1 |
| ES | Linhares | 7 | 350228.9 | 170364 | 31.7249 | 68.3168 | 100 | 34 | 50 | 1 |
| ES | Marechal Floriano | 49 | 28539.72 | 16464 | 68.6302 | 83.8799 | 89 | 40.31 | 0 | 1 |
| ES | Marilândia | 4 | 30894.93 | 12700 | 38.4462 | 72.2643 | 100 | 26 | 66.67 | 1 |
| ES | Muniz Freire | 9 | 67988.7 | 17613 | 38.3388 | 84.3383 | 100 | 57.6 | 0 | 1 |
| ES | Pancas | 19 | 83004.66 | 23059 | 25.8729 | 73.9625 | 100 | 58 | 28.57 | 1 |
| ES | Santa Leopoldina | 58 | 71799.12 | 12300 | 61.5954 | 85.7768 | 72.97 | 42.89 | 33.33 | 1 |
| ES | Santa Maria de Jetibá | 84 | 73562.13 | 39849 | 57.5335 | 90.0573 | 78.58 | 47.11 | 91.43 | 1 |
| ES | Santa Teresa | 62 | 68311.17 | 23392 | 52.0772 | 92.0472 | 88.89 | 51 | 43.4 | 1 |
| ES | São Mateus | 4 | 233753 | 128542 | 36.6549 | 60.5804 | 0 | 17.5 | 100 | 1 |
| ES | São Roque do Canaã | 7 | 34197.39 | 12318 | 37.9574 | 106.3678 | 60 | 51.15 | 0 | 1 |
| ES | Serra | 34 | 55149.48 | 507598 | 35.0568 | 85.1758 | 72.73 | 32.8 | 78.26 | 1 |
| ES | Vargem Alta | 13 | 41374.44 | 21207 | 55.9115 | 84.5863 | 66.67 | 33 | 0 | 1 |
| ES | Venda Nova do Imigrante | 60 | 185999.9 | 24800 | 66.4897 | 83.8152 | 33.33 | 47 | 68.42 | 1 |
| ES | Viana | 23 | 31270.14 | 76954 | 26.3345 | 58.3055 | 60 | 52.2 | 27.78 | 1 |
| ES | Vitória | 12 | 8799.21 | 358267 | 27.7532 | 44.046 | 50 | 60 | 0 | 1 |
| DF | Brasília | 25 | 578832.2 | 2974703 | 15.0703 | 46.7926 | 100 | 36 | 95.24 | 0 |
